# CPP2-p16MIS treatment–induced colon carcinoma cell death in vitro and prolonged lifespan of tumor-bearing mice

**DOI:** 10.1186/s12885-016-2498-4

**Published:** 2016-08-02

**Authors:** Lifeng Wang, Haijin Chen, Jinlong Yu, Xiaohua Lin, Jia Qi, Chunhui Cui, Lang Xie, Shuxin Huang

**Affiliations:** 1 Department of General Surgery, Zhu Jiang Hospital of Southern Medical University, Guang Zhou, China; 2 Department of Ophthalmology, Zhu Jiang Hospital of Southern Medical University, Guang Zhou, China

**Keywords:** Cell-penetrating peptides, Colon cancer, Targeting delivery, CPP2, Molecular therapy

## Abstract

**Background:**

Cell-penetrating peptides (CPPs) are a research hotspot due to their noninvasive delivery ability. Among the identified CPPs, the TAT and R8 peptides have been preferentially applied to transduction into different cells. However, this process is nonselective among various cells. Recent research suggested that CPP2 could selectively penetrate human colorectal cancer (CRC) cells.

**Methods:**

Using in vitro experiments, the mean fluorescence intensity of fluorescein isothiocyanate–labeled CPPs (CPPs-FITC) incubated with different cell lines was compared to corroborate the colon tumor targeting ability of CPP2. The targeting ability of CPP2 was determined in the same way in tumor-bearing mice. We synthesized antitumor peptides by fusing CPP2 to the minimal inhibitory sequence of p16 (p16MIS), which had the ability to restore the function of lost p16, the expression of which was absent in tumor cell lines of various origins. The antitumor effect of the combined peptide was tested in both CRC cell lines and tumor-bearing mice.

**Results:**

In each CRC cell line, the mean fluorescence intensity of CPP2-FITC was higher than that of the TAT-FITC (*p* < 0.001) and R8-FITC (*p* < 0.001) groups. CPP2-p16MIS, the targeting carrier, showed a higher antitumor response in the in vitro cell research. CPP2-p16MIS showed a prolonged mean lifespan of tumor-bearing mice, further characterizing its role in specific tumor-targeting ability in vivo. Survival analysis showed that the mice treated with CPP2-p16MIS had significantly longer survival than the mice treated with phosphate-buffered saline (*p* < 0.05) or those treated with control peptides, including the CPP2 (*p* < 0.05) and p16MIS (*p* < 0.05) groups.

**Conclusion:**

CPP2 could more selectively penetrate CRC cells than TAT or R8 as well as effectively deliver the p16MIS to the tumor.

## Background

Peptides that consist of several cellular or viral proteins were recently determined able to readily penetrate cellular plasma membranes. Because of the specialty of noninvasive and negligibly cytotoxic transport through macropinocytosis and/or endocytosis, they were named cell-penetrating peptides (CPPs) [1, 2]. Accordingly, CPPs have significant biomedical potential as ideal molecular delivery agents. To date, CPP sequences have been used to generate CPP–molecular fusion, leading to the development of cell-permeable peptides/proteins [3], nucleic acids [4], small interfering RNAs [5], peptide nucleic acids [6], small-molecule therapeutics [7], quantum dots [8], and magnetic resonance imaging contrast agents [9]. For example, TAT peptide, one of the earliest identified CPPs, has been heavily utilized for its high solubility and diverse target range [10, 11]. The R8 peptide could also non-selectively penetrate all neoplastic and non-neoplastic cells. For these reasons, here we used TAT and R8 as non-selectively permeable CPP controls.

Although CPPs have been widely used to enhance the intracellular delivery of various cargoes and nanoparticles [12–14], the poor selection between neoplastic and non-neoplastic cells restricted the in vivo application of the CPPs identified to date [15]. CPP cell specificity is especially important for cancer therapy to minimize side effects on normal cells [16]. Therefore, the development of non-toxic, cancer-specific CPPs for effective cancer treatments is greatly needed. Kondo, E et al. [17] recently identified several tumor lineage–homing CPPs, among which CPP2 could more easily penetrate LoVo human colon adenocarcinoma cells than other tumor cell lines. To gain insight into the targeting ability of CPP2 in CRC, HCT116, SW480, and HL-7702 cells were used for comparison with LoVo cells in the in vitro experiments.

Moreover, to investigate whether the in vitro findings were consistent with in vivo findings, fluorescein isothiocyanate (FITC)-labeled CPP delivery was examined in a mouse model of xenograft human colon tumor cells. We found that it was possible for CPP2 to target colon tumor cells and investigated its potential use in peptide-based molecule delivery systems. We subsequently attempted to develop a colon tumor–selective molecular delivery system by leveraging CPP2. Many studies have shown that p16 mRNA expression was absent in tumor cell lines of various origins. Loss of p16^INK4a^ expression is also verified in many different tumors [18–20]. Based on these results, we synthetized an antitumor peptide by fusing CPP2 to the minimal inhibitory sequence of p16 (p16MIS), which had the ability to restore the lost p16 function via the GPG sequence [21–23], and generated a CPP2-fusion peptide for targeting CRC cells.

## Methods

### Materials

The peptides used in this study were purchased from Shanghai GL Biochem Corporation Ltd. (Shanghai, China). RPMI 1640 medium, trypsin-ethylenediaminetetraacetic acid, fetal bovine serum (FBS), penicillin, and streptomycin were purchased from Gibco Life Technologies (Grand Island, NY, USA), while 4′,6-diamidino-2-phenylindole (DAPI) was obtained from Roche (Basel, Switzerland). The experiments were performed in thirty-two 4-week-old BALB/c-nude mice provided by the Animal Experimental Center of Southern Medical University. The experimental animals were selected through ethical approval. The study protocol was approved by the ethics committee of Zhujiang hospital of Southern Medical University.

### Cell lines

HCT116, SW480, and LoVo cells were obtained from the American Type Culture Collection, which performed the cell line authentication using DNA fingerprinting by short tandem repeat analysis. The HL-7702 normal human hepatocyte cell line was purchased from cell bank of the Chinese Academy of Sciences (Shanghai, China), which performed cell line authentication using the DNA fingerprinting method by monitoring the DXS52, Apo-B, MD17S5, and D2S44 primers.

### Cell viability

Each CPP’s toxicity in each cell line was determined by trypan blue dye exclusion. The reduction in viable cell number was examined at the indicated intervals. Cells were loaded on an automatic cell counter (IC1000; Countstar, Shanghai, China), and viable cell number was assessed according to trypan blue dye exclusion.

### Peptide synthesis

The amino acid peptide sequences were as follows: CPP2, DSLKSYWYLQKFSWR; TAT, GRKKRRQRRRPQ; R8, RRRRRRRR; P16MIS, GPGFLDTLVVLHRGPRRRR; and CPP2-p16MIS, DSLKSYWYLQKFSWRGPGFLDTLVVLHRGPRRRR. Here to increase the solubility of CPP2-p16MIS, four arginine residues were added to the C-terminus of each peptide via GP spacers. The chemical solid phase peptide synthesis method was used to synthesize the peptides as follows: 1. Dissolve proper rink amide 4-methylbenzhydrylamine resin with dichloromethane (DCM) and then wash with dimethylformamide (DMF). 2. Wipe off Fmoc- using hexahydropyridine. 3. Add the first amino acid (AA) Fmoc-AA(trt)-OH to O-(benzotriazol-1-yl)-N,N,N′,N′-tetramethyluronium tetrafluoroborate and N,N-diisopropylethylamine, checking the resin after 1 h of reaction time. 4. Discard the solution from Step 3 and wash with DMF. 5. Repeat Steps 2–4 until the last AA is produced and FITC is conjugated with each peptide in the N terminal. 6. Dry the resin after three washes with DCM, methyl alcohol, and DMF. 7. Rinse off the resin and protecting group with lysate solution and then collect the filter liquor and precipitate it by mixing with diethyl ether. 8. Purify the target peptide using high performance liquid chromatography until the purity quotient of each peptide is >95 %.

### Cell penetration assay

The specific targeting ability of CPP2 was found in LoVo cells as previously described [17]. However, it remains unclear whether CPP2 still has this selective permeability in other tumor cell lines of the same origin. To further confirm this point in colon tumor cell lines, several tumor lines, including SW480 and HCT116 cells, were analyzed in this experiment. HL-7702 and LoVo cells were incubated as negative and positive controls, respectively, while TAT and R8 were used for comparison with CPP2.

**Fig. 1:**
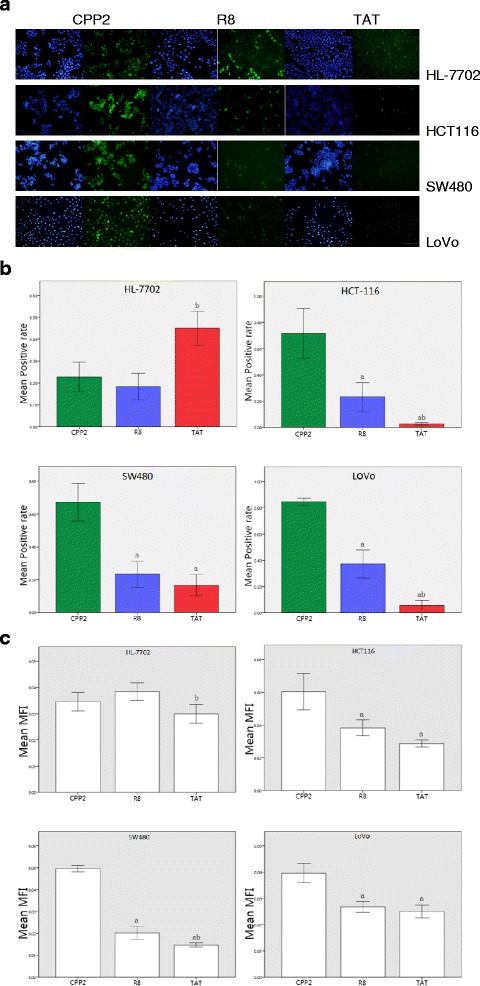
**a** Fluorescence images of three representative cell-penetrating peptides (CPPs) in the different cell lines. Scale bar, 10 μm (amplification factor, ×200). **b** Comparisons of the positive rates of the cell lines showed that CPP2, a colon cancer line–homing peptide, had a higher mean positive rate than that of R8 or TAT (*p* < 0.05). ^a^Significantly different from CPP2 (*p* ≤ 0.05); ^b^significantly different from R8 (*p* ≤ 0.05). **c** Statistical analysis showed that CPP2 had higher mean fluorescence intensity than both R8 (*p* < 0.05) and TAT (*p* < 0.05) in LoVo, SW480, and HCT116 cells. Comparisons of CPP2 vs R8 and CPP2 vs TAT showed no statistical significance in HL-7702 cells (*p* > 0.05). ^a^Significantly different from CPP2 (*p* ≤ 0.05); ^b^significantly different from R8 (*p* ≤ 0.05)

Each cell line was seeded into six-well plates at 2.0 × 10^5^ per well in triplicate and grown in 2.5 mL of growth medium (90 % RPMI 1640 medium, 10 % FBS) before the CPP penetration test. The cells were incubated at 37 °C for 24 h and the permeation tests were conducted at approximately 80 % confluence. The culture medium was replaced with 2.5 mL of mixed growth medium containing 0.3 μM FITC-labeled peptides as previously described. The FITC-labeled peptides were first dissolved in phosphate-buffered saline (PBS) and then added to the RPMI 1640 medium with FBS. The cells were incubated with complexes for 12 h at 37 °C, followed by sufficient washing with PBS to clean the extracellular FITC-CPPs. The cell nucleus was dyed with DAPI which was then discarded, and the cells were washed thoroughly with PBS. Each cell line was examined under an inverted fluorescence microscope (IX71; OLYMPUS, Tokyo, Japan). Five areas were chosen and photographed randomly under the microscopic vision fields (Fig. 1a). Mean fluorescence intensity (MFI) and the positive area of each picture was calculated by using Image J software (National Institutes of Health, Maryland, USA). “Positive rate” here means fluorescein isothiocyanate (FITC) area/4′,6-diamidino-2-phenylindole (DAPI) area in the figure.

### In vivo fluorescence detection

The in vivo distribution of three FITC-labeled CPPs was examined 3 h after intraperitoneal injection into tumor-bearing mice. The tumor mass growing on the nude mice was examined using an in vivo imaging system (Kodak in Vivo Imaging System F; Rochester, New York, USA) to detect the accumulation of the FITC-labeled peptides.

### Survival analysis

Twenty 5-week-old male mice inoculated with SW480 cells were randomly divided into four groups. Two weeks after the inoculation, CPP2-p16MIS, CPP2, p16MIS, and PBS were abdominally administered accordingly. The peptide treatment process was divided into three courses with a 3-day interval between each. In each course, CPP2-p16MIS and its counterparts were administered intraperitoneally every 8 h into the corresponding mice at a dosage of 100 μg per mouse.

### Statistical analysis

The data are shown as $\overline{x}$*± s*. SPSS 21.0 statistical software was used to process the data using one-way analysis of variance, while Tukey’s honest significant differences method was applied to multiple compare among groups and Kaplan-Meier analysis was used for the survival analysis.

## Results

### In vitro fluorescence peptide penetration assay

The intracellular incorporation efficiency of each CPP was semi-quantitatively measured by the detection of mean fluorescence intensity against the background and positive rate. Comparing the mean fluorescence intensities and positive rates of three CRC cell lines, we concluded that CPP2 a higher MFI and positive rate than both R8 and TAT (*p* < 0.05). Comparison of the MFI in HL-7702 revealed that neither CPP2 vs R8 nor CPP2 vs TAT showed any statistically significant difference (Fig. 1c). Figures 1b even showed the positive rate of CPP2 in HL-7702 is not higher than R8 (*p* > 0.05) and lower than TAT (*p* < 0.05). The experimental findings indicated that CPP2 was preferentially internalized by colon adenocarcinoma cells, while R8 and TAT permeated tumor cells relatively poorly by comparison and were preferentially taken in by HL-7702 (Fig. 1a–c).

### In vitro antitumor function test

**Fig. 2:**
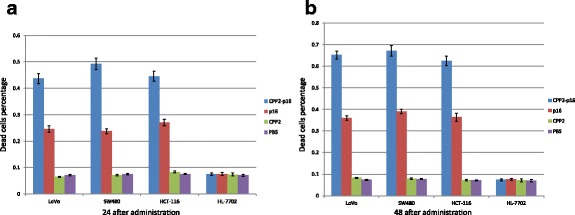
In vitro anti-tumor activity test of cell-penetrating peptide 2 (CPP2)-fusion peptide. **a** The percentage of dead cells in each cell line was calculated after treatment with four different agents 24 h after administration. ^a^Significantly different from CPP2 (*p* ≤ 0.05); ^b^significantly different from R8 (*p* ≤ 0.05); and ^c^significantly different from p16MIS (*p* ≤ 0.05). **b** The percentage of dead cells in each cell line was calculated after treatment with four different agents 48 h after administration. ^a^Significantly different from CPP2 (*p* ≤ 0.05); ^b^significantly different from R8 (*p* ≤ 0.05); and ^c^significantly different from p16MIS (*p* ≤ 0.05)

Antitumor peptides (CPP2-p16MIS) were designed by fusing CPP2, a cell-penetrating domain, to the p16MIS to restore the lost p16 function via the GPG sequence. We explored the anti-tumor function of the synthetic peptides in CRC cells by comparison with p16MIS and CPP2. In three repeat experiments, CPP2-p16MIS had higher cytocidal effects than both CPP2 (*p* < 0.05) and p16MIS (*p* < 0.05). CRC cells treated with CPP2-p16MIS showed maximal cytocidal effects (approximately 45 % of the cells were dead 24 h after transduction with the 10 μM peptide). However, the efficiency of p16MIS is greatly reduced since its lacks the CPP2 portion. As a transduction domain, CPP2 itself did not demonstrate cellular toxicity at the same dose. As for the non-neoplastic HL-7702 cells, none of the three peptides significantly affected their viability at tumor cell suppression doses. This might be correlated with the fact that the p16 protein could be endogenously expressed by HL-7702. Following a single treatment, approximately 63 % of the cells treated by 10 μM CPP2-p16MIS died at 48 h post-introduction, so we speculated the peptide’s effect might maintain for at least a few days (Fig. 2a). In a nutshell, the results showed that the CPP2-p16MIS peptide, which combined the merits of CPP2 and p16MIS, could successfully permeate CRC cells and induce CRC cell apoptosis. Moreover, these data also suggest that CPP2 would elevate the antitumor efficiency of p16MIS in particular tumor lineages.

### In vivo potential peptide complex application research

**Fig. 3:**
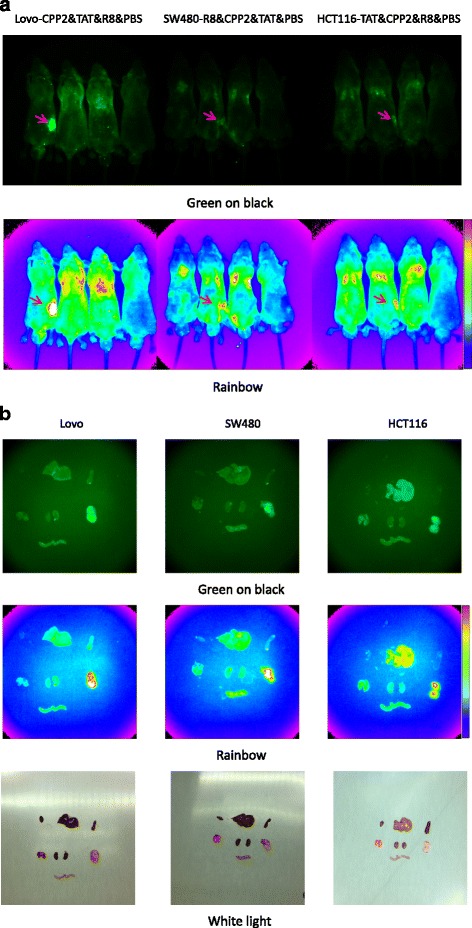
**a** In vivo fluorescence imaging of three representative cell-penetrating peptides (CPPs) and phosphate-buffered saline are displayed by tumor cell line 3 h after the injection of 300 μg of CPPs into nude mice. The tumor mass (red arrow) demonstrated that CPP2 had high selectivity for colorectal cancer cells. R8 and TAT were employed here as representative non-selectively permeable CPP. Green on black and rainbow were the two different capture modes. In the rainbow mode, signal intensity here also meant fluorescence intensity, which was reflected by diverse colors: red > orange > yellow > green > blue (pictured at right). **b** The tumor mass and organs were excised 3 h after the intraperitoneal injection of the CPPs. The heart, liver, and spleen are arranged in the first row, the lungs, kidneys, and tumor mass are shown in the second row, and a part of the small intestine is shown in the last row. Fluorescence imaging of a variety of organs further verified the in vivo imaging results

To further explore the potential in vivo application of these peptides, we primarily compared the targeting ability of the peptides in orthotopically transplanted models using athymic nude mice. In the first phase, twelve 4-week-old male mice were randomly divided into three groups by inoculated cell type. Next, each nude mouse was subcutaneously inoculated with 5 × 10^6^ CRC cells. Fourteen days post-transplant, the in vivo distribution of three FITC-labeled CPPs was examined 3 h after injection. The tumor mass growing on each nude mouse was detected under the in vivo imaging system because of the accumulation of the FITC peptides. In the model mice injected with FITC-CPPs, the fluorescent signal from neoplasms derived from FITC-labeled CPP2 was more intense than that from TAT or R8. Transection of the cancer mass showed that CPP2 tended to selectively penetrate the tumor. We examined the in vivo distribution of the fluorescent CPP2 and compared the fluorescence intensity of the different regions with that of the tumor focus in CRC cell–implanted mice. The uptake of intraperitoneally injected CPP2 in the different body regions was relatively lower than that of the tumor lesions. Mice injected with TAT and R8 mainly had hotspots around their lungs (Fig. 3a). The preferential uptake of CPP2 by the target tumors was further confirmed with fluorescence images of the tumor mass and various normal organs, including the heart, lungs, liver, spleen, intestine, and kidneys (Fig. 3b).

**Fig. 4:**
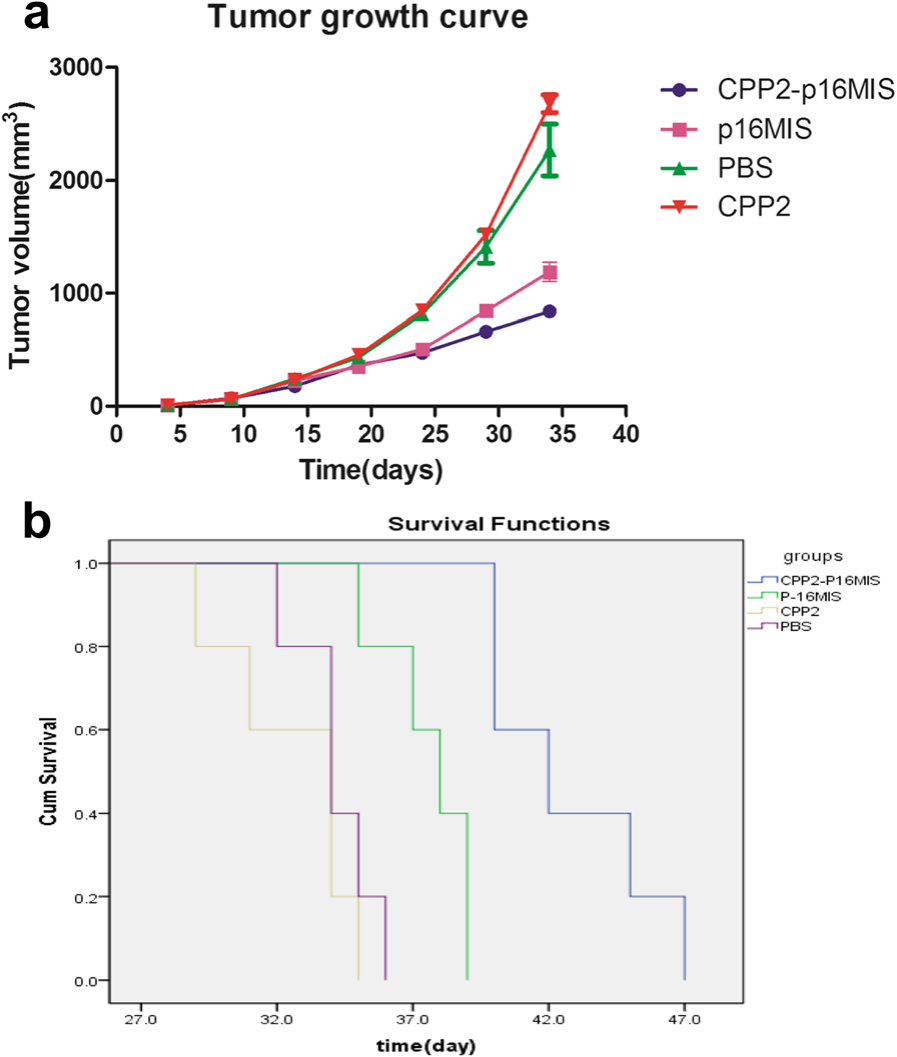
**a** Tumor growth curve in each group. Tumor volumes were calculated using the equation V (mm^3^) = A × B^2^/2, where A is the largest diameter, and B is the shortest axis. **b** Survival analysis results. CPP2-p16MIS had a significantly longer survival time than PBS-treated mice or those treated with control peptides (both CPP2 and p16MIS)

In the mouse model, tumor size did not differ significantly among groups 14 days post-transplantation. At day 24 after inoculation, mice injected with CPP2 and TAT had larger tumors compared to CPP2-p16MIS and p16MIS. Tumor growth curves showed that tumors grew faster in the CPP2 and TAT group. At 29 days after inoculation, mice injected with CPP2-p16MIS had smaller tumors and tumors in this group grew slowly (Fig. 4a). As previously described, the CPP2-p16MIS peptide could successfully permeate CRC cells and induce CRC cell apoptosis. Based on these results, we tested the potential therapeutic utility of CPP2 in vivo. By day 34 after the abdominal administration of CPP2-p16MIS, CPP2, p16MIS, and PBS, most of the mice in the control group (CPP2 or PBS only) had died of aggressive tumors, while the mice treated with CPP2-p16MIS showed no significant cachexia at this time point. Survival analysis (Fig. 4b) showed that the mice treated with CPP2-p16MIS had a significantly longer survival (mean survival [ms], 42.8 days) than PBS-treated mice (ms, 34.2 days) (*p* < 0.005) or mice treated with control peptides, including both CPP2 (ms, 32.6 days) and p16MIS (ms, 37.6 days) (*p* < 0.005).

## Discussion and conclusion

Here we described a kind of CRC-targeting peptide. CPP2 has the potential to preferentially penetrate CRC cells. The permeable process shows no significant cytotoxicity, which means that CPP2 has the capacity to be used as an in vivo molecular vehicle. In vivo research, CPP2 did have the ability to preferentially accumulate in colonic tumor lesions in sharp contrast to the normal organs. A well studied protein p16, which had an antitumor potential, here were used to create an antitumor peptides with CPP2. Compared with CPP2 and p16MIS, CPP2-p16MIS has the advantages of non-cytotoxic delivery as well as antitumor properties in targeted CRC therapy which was confirmed in cell experiments. CPPs as an emerging biomedical delivery system have been highly anticipated since they can translocate across biological membranes and are capable of transporting their cargo inside live cells with minimal invasiveness [24]. HIV-1 transcriptional activator TAT protein [10], the Antennapedia homeodomain of *Drosophila* [25], and poly-arginine (*n* = 4–16) [22, 26] have been the most widely studied CPPs with respect to enhancing the intracellular delivery of CPP-conjugated molecules. Non-selective internalization of CPPs into various cells is the limiting factor for cell-type or tissue-specific targeting applications such as cancer treatments [11]. Therefore, the development of a target-specific drug delivery system is a primary concern for improving the therapeutic efficacy of drugs while reducing their effective doses and side effects [27]. Van Duijnhoven et al. [28] found that radiolabeled activatable CPPs can detect matrix metalloproteinase activity within tumors. Some studies also found a few other carriers for drug delivery [27, 29]. Accordingly, many more experiments are needed to corroborate some special CPPs’ ultimate targeting ability. Here we described the CPP2 structure and demonstrated its unique function of targeting CRC cells, but its mechanism remains unknown. In vivo experiments showed mice injected with TAT-FITC and R8-FITC mainly had hotspots around their lungs. This phenomenon might be correlated with the physical structure of the lungs. Abundant CPPs absorbed into the blood gathered in the lung area because of the capillary network that caused the blood to flow relatively slowly. In addition, we also observed a lot of CPP uptake in the liver in HCT116 inoculated mice. Whether different CPPs had different metabolic time in liver still needs more study. The special ability of CPP2 might offer a new thought to CRC diagnosis and treatment. It may also lead to targeted anticancer therapy in the near future, but the only way we will realize this beautiful dream is for everyone to put their shoulders to the wheel.

## Abbreviations

CPPs, cell-penetrating peptides; CRC, colorectal cancer; CPPs-FITC, fluorescein isothiocyanate–labeled CPPs; DAPI, 4′,6-diamidino-2-phenylindole; FBS, fetal bovine serum; p16MIS, minimal inhibitory sequence of p16; PBS, phosphate-buffered saline
